# Remodelado reverso de aurícula izquierda posablación de fibrilación auricular

**DOI:** 10.47487/apcyccv.v4i2.280

**Published:** 2023-06-30

**Authors:** Diego Xavier Chango Azanza, Cristina Tenorio, Xavier Picón, Jorge Coello, Jessica Robles, Javier Pinos

**Affiliations:** 1 Servicio de Cardiología, Hospital del Rio, Cuenca, Ecuador. Servicio de Cardiología Hospital del Rio Cuenca Ecuador; 2 Servicio de Cardiología, Cardiológico del Austro, Cuenca, Ecuador. Servicio de Cardiología Cardiológico del Austro Cuenca Ecuador; 3 Servicio de Clínica Médica, Clínica Santa Ana, Cuenca, Ecuador Servicio de Clínica Médica Clínica Santa Ana Cuenca Ecuador

**Keywords:** Fibrilación Auricular, Insuficiencia Cardiaca, Ecocardiografía, Ablación por Catéter, Atrial Fibrillation, Heart Failure, Echocardiography, Catheter Ablation

## Abstract

La fibrilación auricular (FA) es la arritmia cardiaca más frecuente. Su asociación con la aparición de eventos embólicos cardiovasculares y de insuficiencia cardiaca es elevada. Los cambios estructurales y funcionales son parte fundamental del proceso fisiopatológico, dando origen a una miopatía auricular izquierda y a disfunción ventricular izquierda progresiva que modifica el pronóstico de los pacientes. Se presenta el caso de un paciente de 75 años de edad con presencia de FA paroxística sintomática en adecuada clase funcional que es derivado para ablación de venas pulmonares posterior a fracaso de la terapia antiarrítmica. El ecocardiograma inicial mostró una función sistólica biventricular conservada, ligera disfunción diastólica y una aurícula izquierda (AI) con volúmenes normales. Sin embargo, se observó deterioro funcional con un *strain* en fase reservorio disminuido. Se realizó aislamiento de las venas pulmonares en forma exitosa sin la evidencia de nuevos eventos arrítmicos, además de una mejoría del *strain* de reservorio de la AI, del *strain* longitudinal global del ventrículo izquierdo (VI) y del índice de trabajo miocárdico a los tres meses de seguimiento. El paciente ha permanecido asintomático y se encuentra bajo seguimiento clínico. El *strain* de AI y VI como nuevas técnicas avanzadas de la ecocardiografía son herramientas útiles en la valoración del remodelado reverso de la miopatía auricular y del daño estructural del VI.

## Abstract (en)

Atrial fibrillation (AF) is the most common cardiac arrhythmia. It has a high association with cardiovascular embolic events and heart failure. Structural and functional changes are a fundamental part of the pathophysiological process, leading to left atrial myopathy and progressive left ventricular dysfunction that modifies the prognosis of patients. We present the case of a 75-year-old patient with symptomatic paroxysmal AF with good functional class who was referred for pulmonary vein ablation after antiarrhythmic therapy failure. The initial echocardiogram showed preserved biventricular systolic function, mild diastolic dysfunction, and normal left atrium (LA) volumes. However, functional LA impairment was observed with decreased reservoir phase strain. Pulmonary vein isolation was successfully performed without evidence of new arrhythmic events, in addition to improvement in LA reservoir strain, left ventricular (LV) global longitudinal strain, and myocardial work index at three months follow-up. The patient has remained asymptomatic and is under clinical follow-up. LA and LV strain as new advanced echocardiography techniques is useful in the assessment of reverse remodeling of atrial myopathy and LV structural assessment.

## Introducción

La fibrilación auricular (FA) es en la actualidad la arritmia cardíaca más frecuente, reportes demuestran que afecta a cerca del 2% de la población en general ^1^. Su presencia está relacionada con la aparición de eventos embólicos cerebrovasculares ^2^, disfunción sistólica del ventrículo izquierdo (VI) con la consiguiente aparición o empeoramiento de la insuficiencia cardíaca (IC) ^3^^,^^4^, disminución en la calidad de vida y una mayor incidencia de muerte de causa cardiovascular ^5^; por lo cual, el diagnóstico temprano es trascendental a fin de evitar desenlaces adversos. 

La estrategia del control de ritmo consiste en restaurar y mantener el ritmo sinusal, en la actualidad constituye la opción de elección, especialmente en etapas iniciales de la enfermedad. Esto puede incluir una combinación de enfoques de tratamiento, incluida la cardioversión eléctrica, medicación antiarrítmica y ablación por catéter, junto con un control de frecuencia adecuado, terapia anticoagulante y terapia profiláctica cardiovascular integral ^1^. En este sentido, la ablación con catéter ha demostrado ser eficaz en mantener el ritmo sinusal en pacientes con FA paroxística y persistente. 

La ablación se recomienda, en general, como terapia de primera línea (IIa) o segunda linea (Ia) después del fracaso (o intolerancia) de los fármacos antiarrítmicos. Esta recomendación se basa en los resultados que muestran la superioridad de la ablación con catéter en relación a la ausencia de arritmia recurrente, la mejoría de los síntomas, la capacidad de ejercicio y la calidad de vida después del fracaso de la medicación con el máximo nivel de recomendación y evidencia científica de acuerdo a las guías actuales (recomendación clase I, nivel de evidencia A) ^1^. 

Por otro lado, la realización de un ecocardiograma transtorácico está indicado en todos los pacientes con diagnóstico reciente de FA, debido a que nos permite evaluar el impacto a nivel anatómico y funcional de la arritmia ^6^. Inicialmente, la evaluación ponía un especial interés en la estructura y tamaño de la aurícula izquierda (AI), la anatomía valvular y la función sistólica y diastólica del VI. Sin embargo, nuevas técnicas de la ecocardiografía, como la deformación miocárdica, han demostrado un valor agregado en la evaluación. El estudio de la deformación miocárdica basado en el seguimiento de marcas (*speckle tracking)* es la técnica actual más utilizada en este sentido. 

Reportamos el impacto en el remodelado reverso de la AI mediante técnicas avanzadas de la ecocardiografía en un estadio precoz de miopatía auricular en un paciente con FA paroxística posterior a la ablación por radiofrecuencia de venas pulmonares mediante la determinación en el seguimiento del valor de *strain* de reservorio de AI. El *strain* de AI es un método novedoso de imagen cardiaca para el estudio de la función y las distintas fases de llenado auricular. El *strain* de reservorio es el parámetro mayormente estudiado y su valor normal en un metaanálisis fue estimado en 39% (IC 38-41) ^7^.

## Reporte de caso

**Figura 1:**
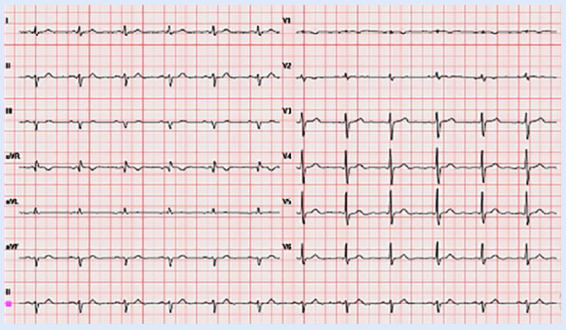
Electrocardiograma basal de 12 derivaciones.

Se presenta el caso de un paciente masculino de 75 años de edad, con antecedente de hipertensión arterial, que es derivado para estudio y seguimiento cardiológico por palpitaciones. Refería antecedente de FA paroxística de 8 meses de evolución, sin tratamiento farmacológico. Presentaba una clase funcional New York Heart Association (NYHA) I. El examen físico cardiovascular fue normal. El electrocardiograma (ECG) basal mostró un ritmo sinusal, signos de crecimiento de aurícula izquierda, QRS angosto con hemibloqueo anterior izquierdo, atraso de conducción por la rama derecha, con repolarización e intervalo QT corregido normal (Figura 1).

**Figura 2:**
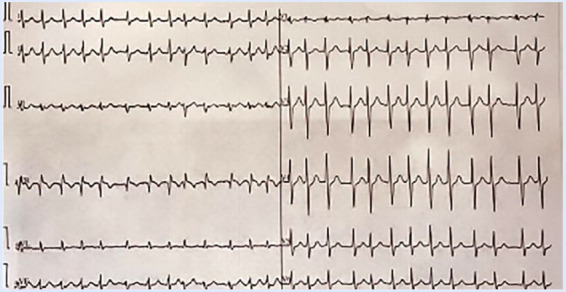
ECG de 12 derivaciones en contexto de palpitaciones mostrando FA paroxística. ECG: electrocardiograma. FA: fibrilación auricular.

Se inicia control de frecuencia a base de 5 mg de bisoprolol una vez al día y control de ritmo a base de propafenona 150 mg cada 12 h. Por presentar un riesgo tromboembólico elevado (*score* CHA2DS2-VASc = 3) y bajo riesgo de sangrado (*score* HASBLED 1), se indica anticoagulación oral (apixaban 5 mg cada 12 h). El paciente presenta a las pocas semanas un nuevo evento arrítmico a pesar del tratamiento instaurado, por lo que acude a sala de emergencias en donde se constata nuevo episodio de FA (Figura 2) que requiere cardioversión farmacológica en forma exitosa.

**Figura 3:**
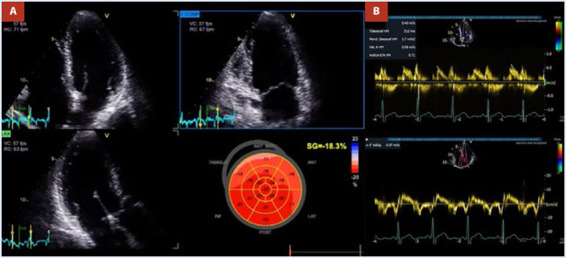
ETT inicial. **A:** Vistas de cuatro, dos y tres cámaras del VI con volúmenes normales y FEVI preservada del 58%. **B:** Patrón de relajación prolongado del VI y relación E/e de 5 en concordancia con ausencia de incremento de las presiones de llenado del VI.

Un ecocardiograma transtorácico (ETT) en ritmo sinusal realizado previamente a la última recurrencia arrítmica mostró tamaño de cavidades normales, función sistólica del VI preservada con fracción de eyección (FEVI) estimada en 58% y un *strain longitudinal global* (SLG) estimado en -18%. Se evidenció una disfunción diastólica con un patrón de relajación prolongado sin signos de incremento de las presiones de llenado del VI, con una relación E/e´ menor de 8. AI con volumen telesistólico normal de 36 mL, (21 mL/m^2^); sin embargo, un *strain* de reservorio disminuido en 18% y sin evidencia de valvulopatías significativas ni datos de hipertensión pulmonar (Figura 3).

**Figura 4:**
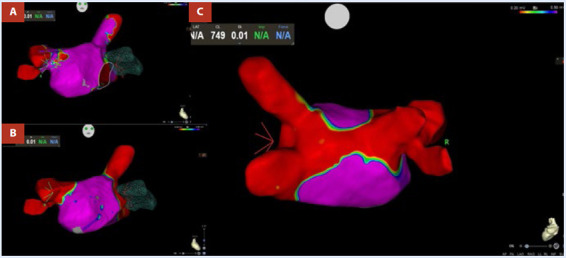
Aislamiento de las VP. **A:** mapa de voltaje de la AI mostrando conexión eléctrica entre VP y AI. **B y C:** mapa de voltaje mostrando desconexión eléctrica entre VP y AI posablación. VP: venas pulmonares. AI: aurícula izquierda.

Como parte de la estrategia de control de ritmo se decide realizar aislamiento de las venas pulmonares, procedimiento que se realiza en forma exitosa. En el mapa de voltaje posablación, se evidenció aislamiento eléctrico de las cuatro venas pulmonares, mostrando bajo voltaje en pared posterior, probablemente secundario al aislamiento antral extenso durante la ablación (Figura 4).

**Figura 5:**
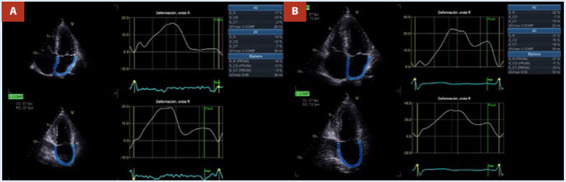
Strain de AI de reservorio comparativo pre y posablación de venas pulmonares. **A:** Vista de cuatro y dos cámaras apical preablación de FA con volumen estimado en 36 mL y *strain* de reservorio estimado en 18%. **B:** vista de cuatro y dos cámaras apical posablación de FA con volumen estimado en 30 mL y *strain* de reservorio de 27%.

El ecocardiograma en el seguimiento a los tres meses posterior a la ablación (periodo de *blanking*) mostró un volumen telesistólico de AI de 30 mL (17,6 mL/m^2^) y una mejoría del *strain* de reservorio de la AI de 27%. Así como una mejoría del SLG del VI y del índice de trabajo miocárdico (ITM) (Figuras 5 y 6). Posterior a concluir la terapia antiarrítmica (propafenona) durante el periodo de *blanking*, el paciente pasó de una clasificación de la Asociación Europea del Ritmo Cardiaco EHRA IIb (síntomas moderados sin afectación de calidad de vida) a EHRA I (sin síntomas) y se ha mantenido en dicha clasificación. Los electrocardiogramas seriados han mostrado ritmo sinusal ^1^.

**Figura 6.**
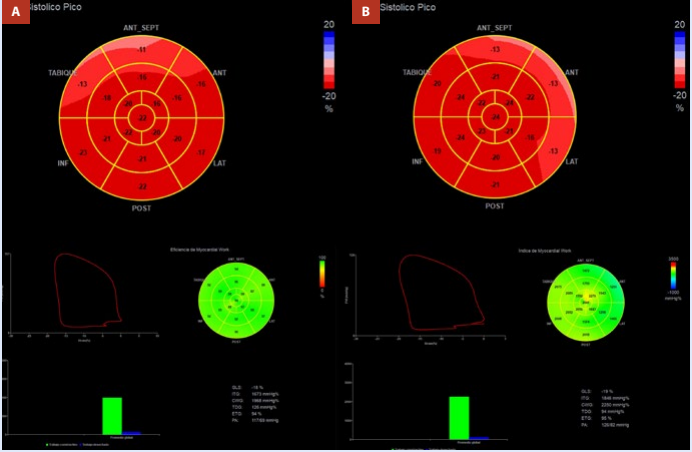
Análisis comparativo de parámetros de eficiencia miocárdica del VI pre y posablación de FA. **A:** SLG del VI preablación de FA con valor de SLG de -18.3%, ITM de 1673 mmHg% y eficiencia de trabajo miocárdico del 94%. **B:** SLG posablación de FA con valor de SLG de -19,9%, ITM de 1846 mmHg% y eficiencia de trabajo miocárdico de 95%.

VI: Ventrículo izquierdo, FA: fibrilación auricular, SLG: Strain longitudinal global, ITM: Indice de trabajo miocárdico.

## Discusión

Recientemente el término «miocardiopatía mediada por FA» se ha instaurado para expresar una importante causa reversible para el desarrollo de IC que se encuentra infradiagnosticada en la práctica cardiológica actual. Esta describe a la FA como la causa de la disfunción ventricular de novo o al empeoramiento de la clase funcional en pacientes con diagnóstico previo. Lo que en la evolución podría causar remodelado estructural y funcional de la aurícula izquierda y disfunción ventricular irreversible si no se trata de forma precoz ^8^. En este sentido, la ablación de FA por radiofrecuencia actualmente es el tratamiento más eficaz para el control del ritmo ^9^, con una tasa de éxito del 60-90% al cabo de dos años en FA paroxística, y del 50-80% en FA persistente, en comparación con el 20 al 40% demostrada con la terapia farmacológica, por lo que constituye la mejor estrategia actual en el control de ritmo.

Las nuevas técnicas de la ecocardiografía mediante el estudio de la deformación miocárdica con el SLG, el *strain* de reservorio de la AI y el ITM por *speckle tracking* han demostrado ser de utilidad para denotar estadios de alteración precoz en distintos escenarios clínicos. La fracción de eyección del VI (FEVI) sigue siendo el parámetro de función sistólica más utilizado en la práctica clínica a pesar de sus limitaciones ^10^. Por otro lado, el SLG del VI ha demostrado ser un marcador sensible de disfunción miocárdica subclínica, dado que pone en evidencia anomalías en la deformación miocárdica en condiciones en donde la FEVI permanece normal ^11^^)^ aunque también sufre de limitaciones al ser dependiente de las condiciones de carga. Recientemente se ha incorporado un método no invasivo para la evaluación del ITM regional mediante el análisis del bucle de deformación tensión/presión del VI mediante ecocardiografía.

Debido a que el ITM incorpora la presión del VI y, por lo tanto, proporciona información incremental sobre la FEVI y la tensión, que son sensibles a la poscarga del VI, y dado que el área del bucle de tensión-presión refleja la demanda metabólica del miocardio y el consumo de oxígeno, este método proporciona información sobre la energía del miocardio utilizada^12^. La evaluación de parámetros como el «trabajo constructivo» analiza los segmentos del VI que se contraen de forma sincronizada y eficiente a fin de contribuir al volumen sistólico durante la contracción del VI. En situaciones patológicas puede existir un alargamiento sistólico de distintos segmentos durante la contracción del VI, condición denominada «trabajo desperdiciado» ya que no contribuye a la eyección del VI. De igual manera, el acortamiento sistólico producido después del cierre de la válvula aórtica (contracción postsistólica) se denomina «trabajo perdido» ya que tampoco contribuye a la contracción eficiente y al volumen sistólico efectivo. Por último, se conoce como «eficiencia del trabajo miocárdico» a la relación entre el trabajo constructivo y la suma del trabajo desperdiciado y constructivo, informada en porcentaje o como la relación con 1 como máximo valor ^12^.

La evaluación de la tensión *strain* de la AI se ha convertido en un método de imagen novedoso con un valor pronóstico superior en comparación con los índices de volumen de la aurícula izquierda y de parámetros de valoración de función diastólica por *Doppler* tisular convencionales. La AI tiene tres funciones, siendo la de reservorio la más importante. Los datos publicados recientemente sugieren el valor pronóstico de la función del reservorio auricular izquierdo en la IC, la FA, el accidente cerebrovascular y la enfermedad cardíaca valvular. Además, la sobrecarga del reservorio auricular izquierdo demostró ser un predictor de morbimortalidad cardiovascular en la población general _^(13)^_ . Este parámetro es estimado por ecocardiografía transtorácica mediante el procesamiento de las curvas de llenado de la AI adquiridas desde vistas de cuatro y dos cámaras apicales. Por último, cabe recalcar que la tensión de la AI depende de las condiciones carga y está fuertemente influenciada por la función del VI, por lo que están claramente relacionadas. 

En nuestro reporte de caso ponemos en evidencia el aporte de estas nuevas técnicas de deformación miocárdica en contexto de ablación por radiofrecuencia de FA. Además de la mejoría sintomática del paciente, la desaparición de los síntomas posterior al procedimiento, a pesar de que el seguimiento es corto y se requiere un mayor tiempo de observación, se relacionó con mejoría de los parámetros en la función de reservorio de la AI, aun sin cambios evidentes en los volúmenes o parámetros de la función diastólica. Además de mejoría de parámetros de la función sistólica sin cambios en la FEVI, como la mejoría en el SLG, en el índice de trabajo miocardio, un menor valor de trabajo miocárdico desperdiciado y a una consiguiente mejor eficiencia del trabajo miocárdico.

En conclusión, las nuevas técnicas de deformación por ecocardiografía permiten poner en evidencia daños estructurales y funcionales subclínicos que pueden ser reversibles en el seguimiento por encima de los parámetros convencionales. El presente caso muestra una mejoría marcada del *strain* de AI en el seguimiento a corto plazo en un paciente posterior a ablación de FA en relación a remodelado reverso, hipotetizando una regresión de la miopatía auricular que puede estar relacionada a mayor tasa de eventos cardiovasculares.
